# Rapid and Sensitive Detection of Antibiotic Resistance Genes by Utilizing TALEs as a Diagnostic Probe with 2D-Nanosheet Graphene Oxide

**DOI:** 10.1021/acs.analchem.3c00647

**Published:** 2023-06-13

**Authors:** Jihye Kang, Van-Thuan Nguyen, Moon-Soo Kim

**Affiliations:** Department of Chemistry, Western Kentucky University, Bowling Green, Kentucky 42101, United State

## Abstract

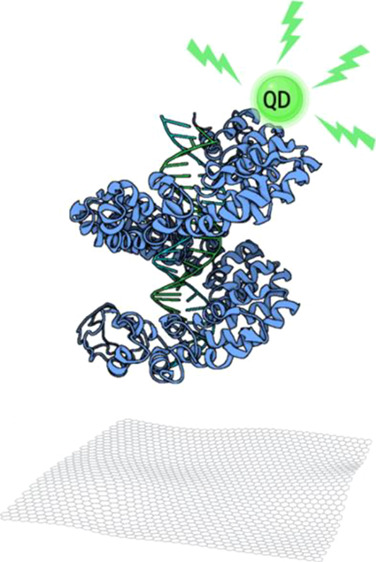

As antibiotic resistance has risen as one of the major health concerns associated with infectious diseases due to the reduced efficacy of antibiotics, rapid and sensitive detection of antibiotic resistance genes is critical for more effective and faster treatment of infectious diseases. A class of programmable DNA-binding domains called transcriptional activator-like effectors (TALEs) provides a novel scaffold for designing versatile DNA-binding proteins due to their modularity and predictability. Here, we developed a simple, rapid, and sensitive system for detecting antibiotic resistance genes by exploring the potential of TALE proteins for the creation of a sequence-specific DNA diagnostic along with 2D-nanosheet graphene oxide (GO). TALEs were engineered to directly recognize the specific double-stranded (ds) DNA sequences present in the tetracycline resistance gene (*tetM*), avoiding the need for dsDNA denaturation and renaturation. We take advantage of the GO as an effective signal quencher to quantum dot (QD)-labeled TALEs for creating a turn-on strategy. QD-labeled TALEs are adsorbed on the GO surface, which will bring QDs in close proximity to GO. Due to the fluorescence quenching ability of GO, QDs are expected to be quenched by GO via fluorescence resonance energy transfer (FRET). QD-labeled TALE binding to the target dsDNA would lead to the conformational change, which would result in dissociation from the GO surface, thereby restoring the fluorescence signal. Our sensing system was able to detect low concentrations of dsDNA sequences in the *tetM* gene after only 10-minute incubation with the DNA, providing a limit of detection as low as 1 fM of *Staphylococcus aureus* genomic DNA. This study demonstrated that our approach of utilizing TALEs as a new diagnostic probe along with GO as a sensing platform can provide a highly sensitive and rapid method for direct detection of the antibiotic resistance gene without requiring DNA amplification or labeling.

Antibiotics are frequently administered to treat bacterial infectious diseases in animals and humans. One of the common antibiotics is tetracycline due to its low toxicity and the broad spectrum of its activity.^1^ The World Health Organization (WHO) reported that tetracycline exhibits the most efficacy in the treatment of cholera and several other clinical trials. However, the efficacy of tetracycline has been reduced due to the steady emergence of antibiotic resistance genes (ARGs).^2^ Resistance to tetracycline is governed by the *tet* genes, which are involved either in the active efflux of the drug, ribosomal protection, or enzymatic drug modification.^3^ The *tet*(M) gene is widely distributed among both gram-positive and gram-negative bacteria.^2^ Moreover, the *tet*(M) determinant is considered to motivate the sole tetracycline resistance mechanism in mycoplasmas, which protects the bacterial ribosome from the effects of antibiotics.^4,5^ The most common method for detecting antibiotic resistance genes uses traditional nucleic acid amplification such as polymerase chain reaction (PCR), which requires multi-step reactions, well-trained personnel, and many expensive reagents with a two-hour assay time.^3,6^ These shortcomings have impeded application in nonlaboratory and limited-resource settings. To overcome such limitations of traditional nucleic acid detection methods, it is crucial to develop new diagnostic tools for screening and detecting antibiotic resistance genes such as simple and rapid point-of-care testing.

A DNA-binding protein can directly read the sequence information from double-stranded DNA (dsDNA), avoiding the need for DNA denaturation and subsequent renaturation with carefully designed probes under controlled conditions.^7−9^ Compared to a zinc finger protein, transcriptional activator-like effectors (TALEs) are a new class of DNA-binding domains, which can provide a novel scaffold for designing versatile DNA-binding proteins due to their modularity in the structure of the central DNA-binding region. TALEs are predominantly secreted from *Xanthomonas* and they function as transcriptional activators of certain plant genes.^7^ TALEs share a common domain organization that enables them to be imported into nuclei and act as transcriptional activators.^10^ The central DNA-binding domain of TALEs consists of multiple tandem repeats with each repeat recognizing one specific DNA base pair. Each repeat region consists of 34 amino acid residues that are nearly identical except for the two amino acids at positions 12 and 13 that are hypervariable.^9,11,12^ These two consecutive polymorphic amino acids are termed repeat variable di-residues (RVDs).^11,12^ TALEs can be designed to bind any desired target DNA sequences by simply assembling the corresponding RVDs for each DNA base in the sequences of target DNA. Thus, engineered TALEs possess high predictability toward target DNA bases and good programmability in designing, which is advantageous. Due to these advantages of TALEs, base-specific recognition by the TALE repeats can greatly facilitate the rational design of novel DNA-binding proteins with a wide range of biomedical and biosensor applications.

Graphene oxide (GO) is a two-dimensional nanosheet, which is known for its possession of a large surface area and excellent biocompatibility with biomolecules such as DNA, cells, antibodies, and other proteins.^13,14^ GO has a largely hydrophobic basal plane with various oxygen-containing functional groups such as epoxides, carbonyl, carboxyl, and hydroxyl groups.^15^ Thus, protein molecules can interact with GO via noncovalent interactions such as electrostatic interaction, hydrogen bonding, hydrophobic interaction, and π – π stacking interaction. However, GO hardly interacts with dsDNA.^13,16−18^ The incorporation of biological molecules with GO has extensive potential in the development of biosensors. In addition, GO can quench the fluorescence via fluorescence resonance energy transfer (FRET).^15,19−25^ The fluorescence signal of fluorophores can be quenched by GO in proximity and the effective quenching distance can be extended to ∼30 nm.^19,26^

As a fluorescent molecule dissociates beyond 30 nm from GO, the signal is expected to be restored and it can then be measured.^14,27,28^ Due to the large size of TALEs relative to fluorescent labeled short single-stranded aptamers, TALEs can more readily dissociate further away from GO beyond 30 nm upon DNA binding, which may result in more restoration signal and higher sensitivity. In this study, we have demonstrated a GO-based sensor with engineered TALEs labeled with quantum dots (QDs) for direct detection of the antibiotic resistance gene (Scheme 1). TALEs are known to alter the conformational characteristic to a compressed helical shape upon DNA binding from an extended helical conformation in the absence of DNA.^8^ This can cause the prompt dissociation of TALEs from GO in the presence of target DNA, resulting in a turn-on signal. Our detection method was able to detect the target DNA sequence as low as 1 pM for oligonucleotides and 1 fM for genomic DNA after 10 min incubation. Therefore, our novel approach of using TALEs as a new diagnostic probe along with GO enables us to develop a rapid, simple, and sensitive method of detecting antibiotic resistance genes by avoiding the laborious steps of DNA denaturation and subsequent hybridization required for the conventional amplification method of nucleic acids.

**Scheme 1:**
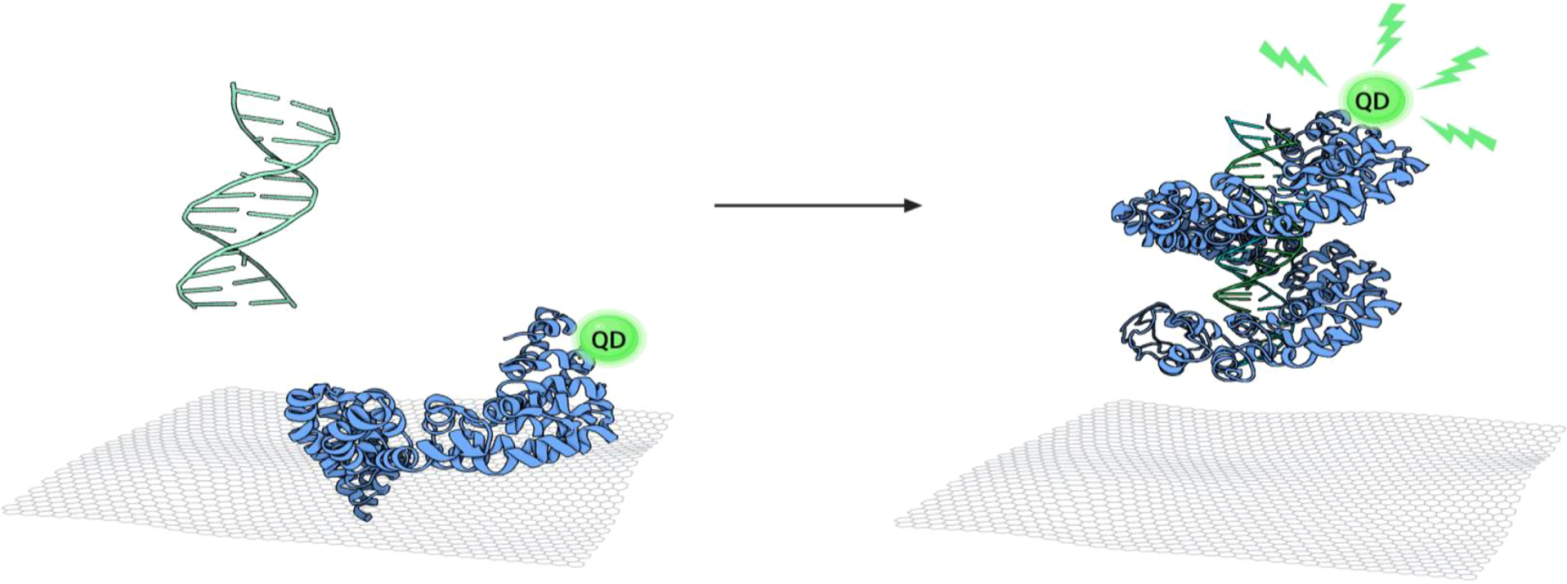
Schematic Representation of TALEs and GO-Based Biosensors for Detecting the Antibiotic Resistance Gene

## Experimental Section

### Construction, Expression, and Purification of TALEs

TALEs were engineered by assembling the corresponding RVDs of TALEs for each DNA base of the target regions in the *tetM* gene. The DNA coding regions for each TALE were commercially synthesized by GenScript. The two TALE proteins tetM_1298 and tetM_611 were subcloned between StuI and AatII sites of the pMAL-c2X vector, replacing engineered AvrBs3 TALE via an infusion cloning kit purchased from Takara. The pMAL-c2X vector contains a maltose-binding protein (MBP) and histidine (His) tag for protein purification purposes. The plasmids were transformed into *Escherichia coli* BL21 and then expressed in *E. coil* after induction of isopropyl ß-D-1-thiogalactopyranoside (IPTG) at an OD_600_ of 0.6–0.8 for 3 h at 37 °C. Cells were pelleted and resuspended in lysis buffer (500 mM NaCl, 20 mM Tris base, 20 mM imidazole, and protease inhibitor at pH 8.0). After sonication, proteins in cell lysates were applied to the nickel resin of a HisTrap column (Cytiva), washed with buffer A (20 mM Tris, 15 mM imidazole, and 1 mM TCEP at pH 8.0) and buffer B (1 M NaCl, 20 mM Tris, 15 mM imidazole, and 1 mM TCEP at pH 8.0), and eluted in elution buffer (250 mM NaCl, 250 mM imidazole, 20 mM Tris, and 1 mM TCEP at pH 8.0) by the ÄKTA-Go system (Cytiva). The concentration and purity were assessed by Coomassie-stained polyacrylamide gel electrophoresis with sodium dodecyl sulfate (SDS-PAGE) and the Bradford assay using bovine serum albumin (BSA) standards. Purified proteins were stored in a buffer (480 mM KCl, 12 mM Tris, 2 mM DTT, 40% glycerol at pH 6.2) at −20 °C until use.

### GO Preparation and Surface Characterization

GO dispersion (ACS Material, Pasadena, CA) was vortexed to ensure a homogeneous solution before diluting with deionized water. A serial dilution was performed for the preparation of different GO concentrations. The stock GO dispersion of 5 mg/mL was initially diluted to 1 mg/mL and subsequently diluted down to 1 μg/mL with 10 μg/mL. The single layer ratio is >80% with the size of GO sheets ranging from 0.5 to 2.0 μm and a thickness of 0–2 nm. The morphology of QD-labeled TALEs adsorbed on the GO surface was measured with transmission electron microscopy (TEM) JEM-1400plus (JEOL, Peabody, MA). The surface topology of QD-labeled TALEs on the GO surface was measured by atomic force microscopy (AFM) with an Agilent 5500 (Agilent, Santa Clara, CA) using contact mode equipped with a silicon AFM tip (PPP-CONTR, Nanosensors).

### Quantum Dot Conjugation on TALEs

Carboxyl PEG functionalized CdSe/ZnS quantum dots in water with an emission peak from 520 to 530 nm (Creative Diagnostics, Shirley, NY) were covalently conjugated with the amine group of TALEs using EDC/NHS chemistry. The 1:2 ratio of the concentration of EDC to NHS was optimized in our previous study.^20^ The molar ratio of QD–TALEs was then optimized by labeling at a set concentration of QD and varying concentrations of TALEs. The molar ratio of QD–TALEs–EDC–NHS was determined to be 1:2:100:200, respectively (refer to the Results and Discussion section). For QD labeling, 30 μL of 2 μM QDs was added to 210 μL of HEPES buffer (100 mM HEPES and 500 mM NaCl at pH 7.5), and 30 μL of 5 μM EDC (Thermo Scientific, Rockford IL) and 30 μL of 10 μM NHS (Thermo Scientific, Rockford, IL) were added to the solution, which was incubated for 20 min at room temperature. Subsequently, 300 μL of 400 nM TALEs was added to the reaction and incubated for 2 h at room temperature. Labeled TALEs were subject to the buffer exchange with 300 μL of TALE storage buffer (480 mM KCl, 12 mM Tris, and 2 mM DTT at pH 6.2) using a 0.5 mL 50 K MWCO ultrafiltration unit (PES filter) (Thermo Scientific, Rockford, IL) and then stored at 4 °C in the dark until use. The concentration of labeled TALEs was determined to be 400 nM as remained in 300 μL of TALE storage buffer.

### TALE Assay with GO

Complementary pairs of forward and reverse oligonucleotides were prepared by heating at 95 °C for 10 min and they were slowly cooled to 55 °C by 1 °C per 40 s and then incubated for 15 min at 55 °C. Subsequently, the oligonucleotides were cooled to 4 °C by 1 °C per 40 s to form the double-stranded DNA. The sequences of oligonucleotides are provided in Figure S1 of the Supporting Information. In a black 96-well plate with a clear flat bottom (Corning, Kennebunk, ME), 10 μL of 100 nM QD-labeled TALEs and 10 μL of 50 μg/mL of GO dispersion were added into 70 μL of TALE storage buffer and then incubated at room temperature for 30 min. Subsequently, 10 μL of the target dsDNA, such as oligonucleotides or genomic DNA, was added and mixed well by gentle tapping and then allowed to incubate for 10 min. The fluorescence intensity and emission spectrum were measured by a Synergy H1 multiplate reader (BioTek Instruments, Winooski, VT). Fluorescence intensity was measured with an excitation wavelength (λ_ex_) of 370 nm and an emission wavelength (λ_em_) of 525 nm. The fluorescence emission spectrum was measured with an λ_ex_ of 370 nm and an λ_em_ from 440 to 600 nm with a 5 nm interval and a gain of 75. All optical measurements were performed at room temperature under ambient conditions. All experiments were repeated in duplicate, and the standard error was calculated from duplicate samples.

The genomic target DNA of *Staphylococcus aureus* (ATCC 700699) containing the *tetM* gene was purchased from ATCC. The genomic DNA (10 ng) was digested by Sau3AI (NEB, Ipswich, MA) by incubating for 1 h at 37 °C. Subsequently, the reaction mixture was incubated for 20 min at 65 °C to inactivate Sau3AI. As the molecular weight of genomic DNA (2.8 × 10^6^ base pairs) is calculated to be 17.5 × 10^8^ g/mol, the molar concentration of 1 fM is equivalent to a weight concentration of 1.8 pg/μL in the final assay volume of 100 μL. The concentrations of genomic target DNA used for the genomic assay were 1 fM and 10 fM which will contain 180 pg and 1.8 ng of digested genomic DNA, respectively.

### Electrophoretic Mobility Shift Assay (EMSA)

Complementary pairs of 5′-biotin labeled forward and reverse oligonucleotides in annealing buffer (10 mM Tris and 500 mM NaCl at pH 7.5) were annealed by heating at 95 °C for 10 min and slowly cooled to 55 °C by 1 °C per 40 s, followed by additional incubation at 55 °C for 15 min. Subsequently, oligonucleotides were cooled to 4 °C by 1 °C per 40 s to form the double-stranded DNA. Binding reactions were performed at room temperature in the dark for 1 h and then at 4 °C for 30 min in EMSA binding buffer containing 12 mM Tris, 60 mM KCl, 2 mM DTT, 0.1 mg/mL BSA, 30% glycerol, 5 mM MgCl_2_, 0.2 mM EDTA, 500 pmol target DNA, and purified TALEs with concentrations ranging from 0.03 to 200 nM. Gel electrophoresis was performed in the cold on a 9% native polyacrylamide gel in 0.5× TBE buffer. After blotting on a nylon membrane by transferring in the cold, the DNA was cross-linked by a UV cross-linker for 4 min. EMSA was performed using the light shift chemiluminescent EMSA Kit (Pierce, Rockford, IL) according to the manufacturer’s protocol. The chemiluminescent signal was read using an AlphaImager HP (ProteinSimple, San Jose, CA).

## Results and Discussion

### Engineering and Purification of TALEs

**Figure 1:**
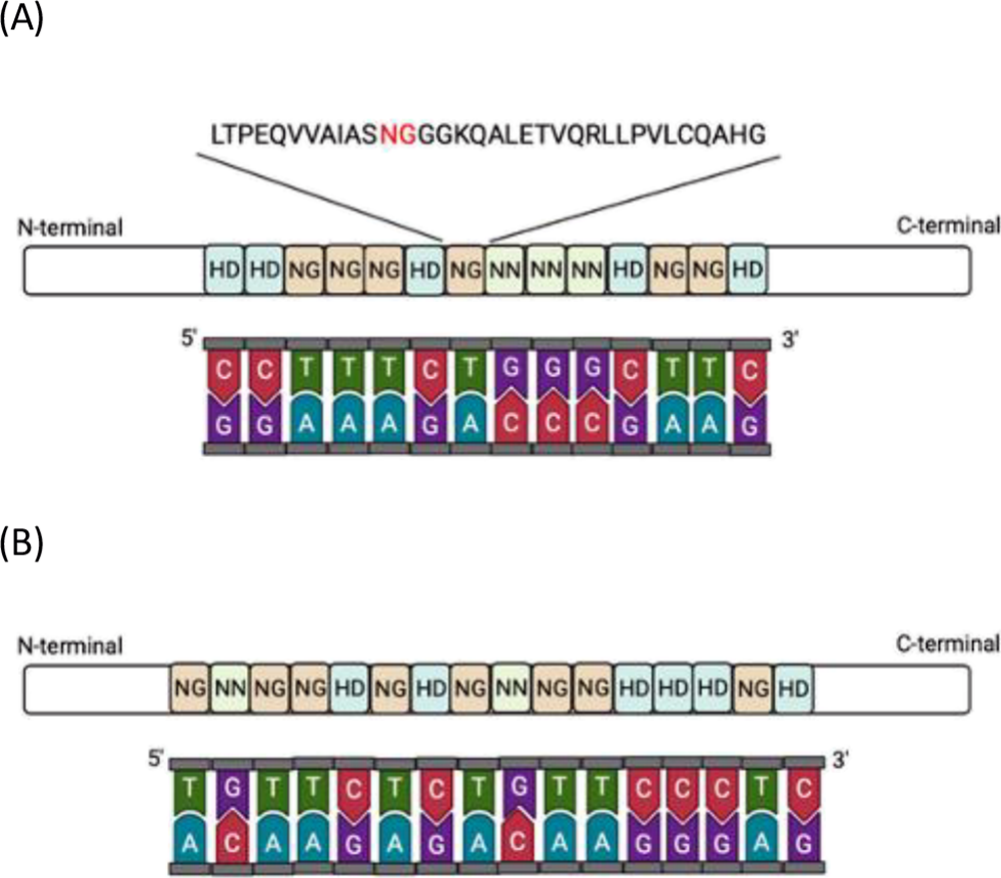
Sequence of RVDs and their respective target DNA sequence. (A) TALE tetM_1298 and (B) TALE tetM_611. HD, NG, NN, and NI RVDs specify C, T, G, and A, respectively.

TALEs were constructed to recognize specific regions of the DNA sequences of the tetracycline resistance gene *tetM* (see the sequences in Figure S2). The TALE tetM_1298 and tetM_611 were designed to recognize the 14 and 16 base pairs within the *tetM* sequence, respectively (Figure 1). In principle, a 12 bp of DNA sequence is long enough to specify a unique site in the bacteria genome as the number of possible combinations of occurrence of 12 bp would be 16.77 × 10^6^, which is bigger than the sizes of the *Staphylococcus aureus* genome (2.82 × 10^6^) and *E. coil* genome (5.44 × 10^6^). Thus, the engineered TALEs would bind unique sites in the *S. aureus* genome. The purity of purified TALEs was evaluated using SDS-PAGE gel showing approximately 95% purity (Figure S1). After IPTG-induced expression and nickel column purification, TALEs were the primary species in the fractions, as shown in the gel (Figure S1). To develop multiplexed detection system, the design of individual TALEs would be required to recognize specific sequences in the respective ARGs.

### Optimization of QD Labeling

**Figure 2:**
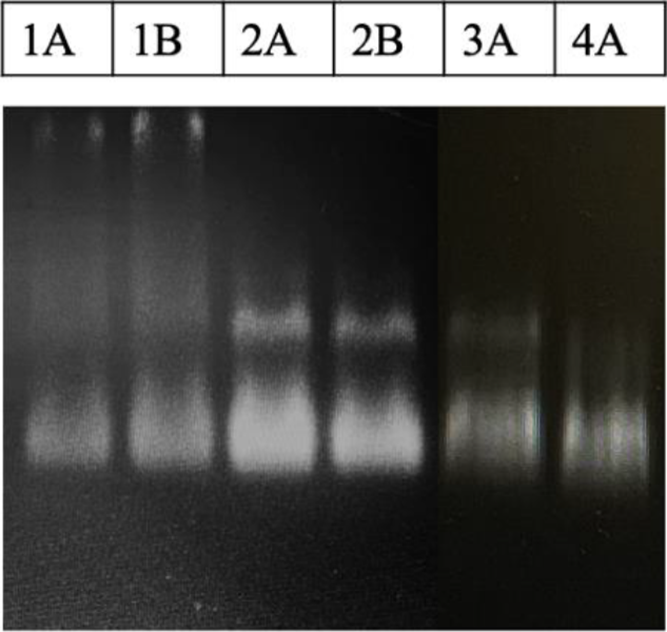
Optimization of QD labeling on TALEs using EDC/NHS coupling chemistry. The molar ratio of QD:TALE:EDC:NHS of (1A) 1:50:100:200, (1B) 1:50:80:1600, (2A) 1:10:100:200, (2B) 1:10:800:1600, (3A) 1:5:100:200, and (4A) 1:2:100:200.

In the presence of an excess of TALEs, the QD-labeled TALEs could be formed in a couple of different species, depending on the number of TALE molecules conjugated on the QD. Thus, the QD labeling of TALEs should be optimized to be able to obtain consistent detection signals resulting from the uniform species of QD-labeled TALEs. The optimal molar ratio of EDC:NHS has already been demonstrated in our previous study.^20^ In addition, the EDC:NHS molar ratio of 1:2 has been previously reported as the most effective ratio for EDC/NHS coupling conjugation in other studies.^20,29^ We thus investigated the molar ratio of QD:EDC. Our result indicated that the molar ratio of QD:EDC does not impact the formation of the uniform species of QD-labeled TALEs (Figure 2). However, the different molar ratios of QD:TALE contributed to variations in the presence of different species of QD-labeled TALEs. As shown in Figure 2, mobility of the labeled species was observed for lanes 1A–3A, indicating that the different species of QD-labeled TALEs were formed as the molar ratio of QD:TALE increased up to 1:50. It is expected that the excess molecules of TALEs could be additionally conjugated to QDs, resulting in the production of a bigger size of QD-labeled TALEs, as shown in lanes 1A–3A of Figure 2, as compared to the formation of one species of labeled TALEs at the 1:2 molar ratio of QD:TALE in lane 4A. Thus, the optimal molar ratio of QD:TALE:EDC:NHS for labeling was determined to be 1:2:100:200 as evidenced by the uniform species of QD-labeled TALEs in lane 4A of Figure 2.

### QD-Labeled TALEs Adsorbed on GO

**Figure 3:**
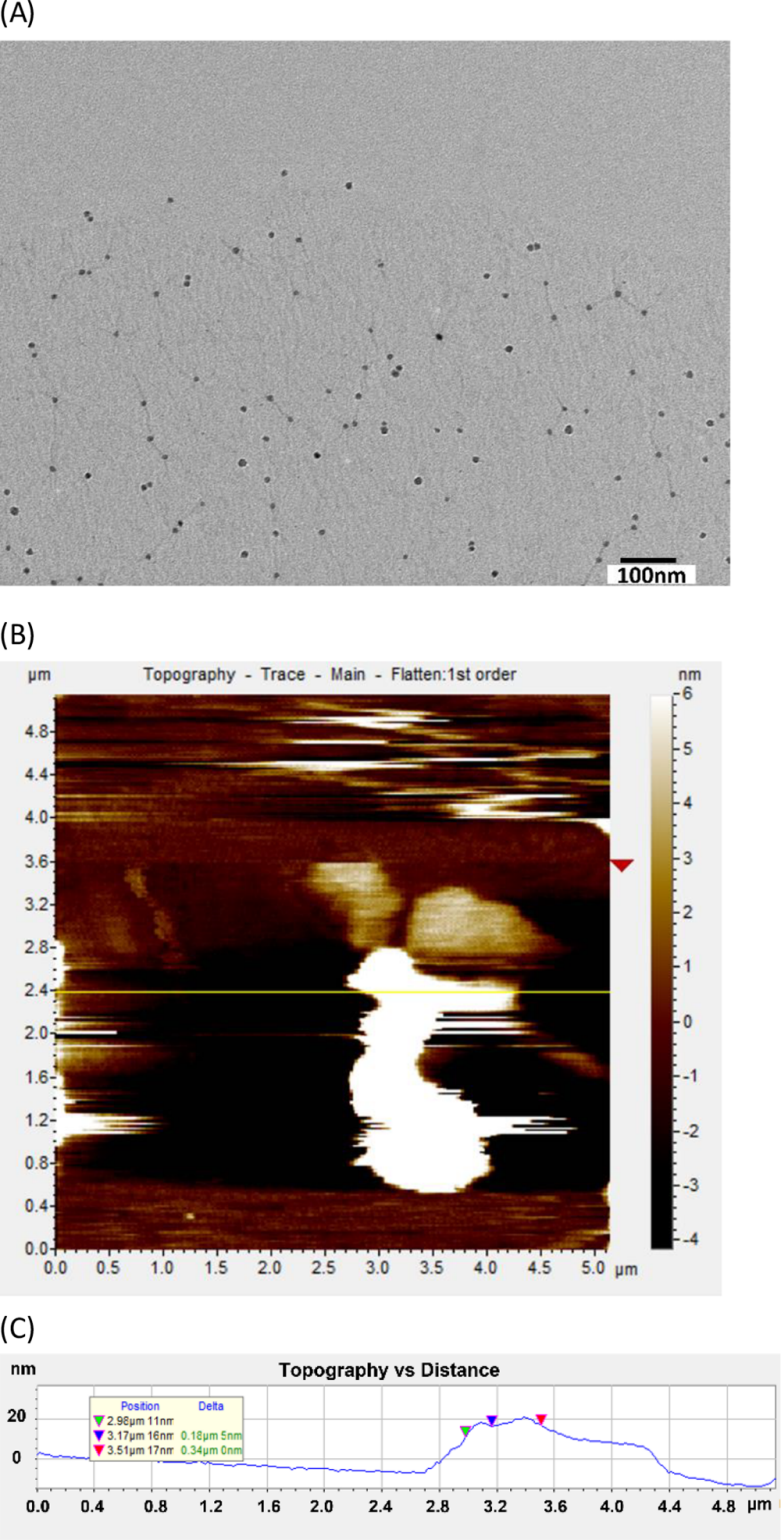
(A) TEM image of QD-labeled TALEs adsorbed on GO, (B) AFM image of the complex of QD-labeled TALEs and GO, and (C) height profile of the corresponding yellow line scan.

The morphology of GO was already evaluated by using a transmission electron microscopy (TEM) JEM-1400plus (JOEL, Peabody, MA, USA) device in our previous study.^20^ The morphology of GO nanosheets is shown to have wrinkles with a >80% single-layer ratio.^20^ The size of GO ranges from 0.5 to 2 μm with a width of 2 nm based on a two-dimensional (2D) lattice.^20^ A key aspect of our detection method is the adsorption of QD-labeled TALEs on the GO surface, resulting in fluorescence quenching of QDs via FRET. However, it has not been demonstrated yet that TALEs can be immobilized on the GO surface. Proteins can adsorb on the GO surface depending upon the various specific and nonspecific interactions between the residues of proteins and the GO surface.^13,18^ Our engineered TALEs are expected to be immobilized on the GO surface via non-covalent interactions such as π – π stacking and electrostatic interactions, given that approximately 7.7% of aromatic side chains, 14.6% of amide side chains, and 5.3% of charged hydrophilic side chains are present in the peptide sequence of the TALEs. (Supporting Information Table S1).^18^ Here, we demonstrated for the first time the adsorption of QD-labeled TALEs onto the GO nanosheets by TEM and AFM. As seen in Figure 3A of the TEM image, the stained QDs of QD-labeled TALEs were adsorbed onto the GO surface and the size of QDs is approximately 10 nm. In addition, the AFM image indicated that the approximate height of QD-labeled TALEs adsorbed on GO is 20 nm (Figure 3B,C). Considering that the sizes of GO, QD, and TALE are 2, 10, and 6 nm, respectively, this result confirmed that QDs-labeled TALEs were adsorbed on the GO nanosheet surface. Thus, the fluorescence signal is expected to be quenched within a distance of 30 nm to GO by FRET.^26^

### Quenching Efficiency of GO

**Figure 4:**
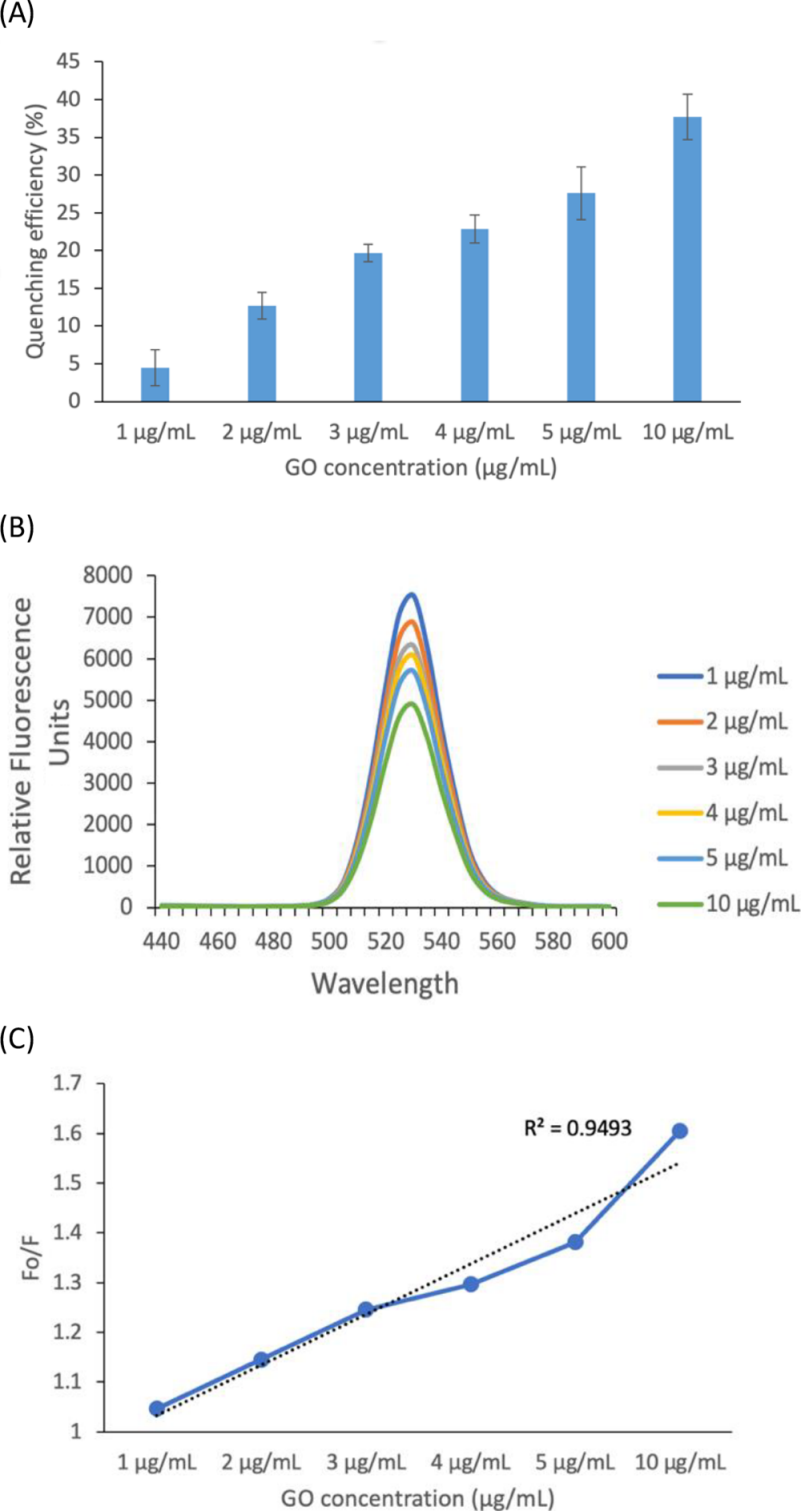
Change in the (A) quenching efficiency, (B) fluorescence spectra of QD-labeled TALEs by increasing the concentration of GO from 1 to 10 μg/mL, and (C) Stern–Volmer plot of QD-labeled TALEs at the different concentrations of GO.

The optimized concentration of GO is necessary to accomplish sensitive detection of our approach as quenching efficiency can affect the generation of the detection signal. For example, lower quenching efficiency can contribute to the background signal, resulting in a reduction of the limit of detection (LOD), whereas a higher quenching efficiency can cause inadequate restoration of fluorescence intensity for target DNA detection due to the oversaturation of GO nanosheets.^20,21,24^ Thus, we evaluated the effect of GO concentrations on the quenching of labeled proteins over the range from 0 to 10 μg/mL of the GO. As shown in Figure 4A, the quenching efficiency was enhanced with increasing concentrations of GO. The previous report indicated that 30% quenching efficiency would be optimal due to the large size of a dye-labeled protein with a size of 150 kDa.^30^ The GO concentration of 5 μg/mL provided approximately 28% quenching efficiency of QD-labeled TALEs, which was in good agreement with this previous report^33^ as our TALEs are also large proteins with a size of 120 kDa. The quenching efficiency of QD-labeled TALEs by GO was calculated using eq 1, where *F*_0_ and *F* are the fluorescence intensity at the maxima in the absence and presence of GO, respectively.^20^
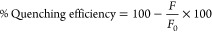
1

In addition, we demonstrated that the restoration of fluorescence signals was solely due to fluorescence intensity from QD-labeled TALEs by measuring the fluorescence emission spectrum ranging from 440 to 600 nm. A sole and strong emission peak was observed at 530 nm from CdSe/ZnS quantum dots (Figure 4B). A linear quenching effect corresponding to the concentrations of GO was demonstrated by a Stern–Volmer plot of *F*_0_/*F* against the concentration of GO, where *F*_0_ and *F* were the fluorescence intensity at the maxima in the absence and presence of GO, respectively (Figure 4C). Based on the data shown in Figure 4, we decided to use 5 μg/mL of GO as the optimal concentration for the rest of our study.

The detection signal turns on when QD-labeled TALEs dissociate from GO by binding to their target DNA sequence. TALEs will associate nucleic acid components to form specific and stable multiunit complexes via hydrogen bonding.^31^ Thus, the interaction between TALEs and their target DNA would be more stable and stronger compared to the electrostatic interaction between TALEs and the GO surface, which would result in the dissociation of TALEs from GO. We believe that the stable TALEs-DNA bound complex would enable a highly sensitive and specific detection system. To investigate TALEs and DNA interactions, the binding affinities (*k*_D_) of the engineered TALEs were measured using electromobility shift assay (EMSA). As shown in Supplementary Figure S2, the binding affinities (*k*_D_) of the TALE tetM_1298 and tetM_611 toward their target DNA sequences were determined to be 1.2 and 4.6 nM, respectively.

### Sensitivity

**Figure 5:**
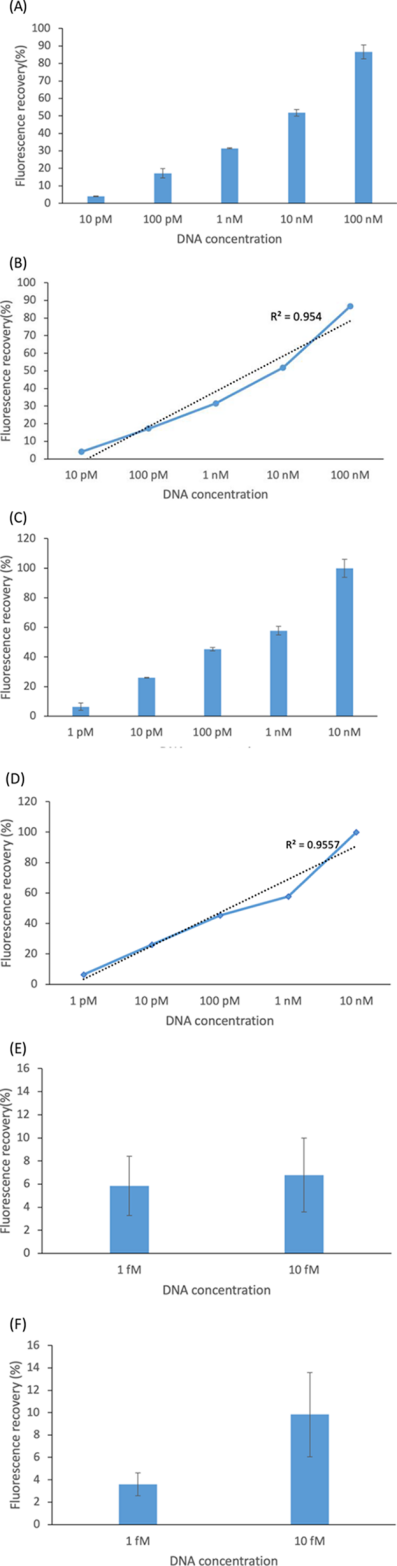
Sensitivity of GO-TALE-based assay. The assay was performed with DNA oligonucleotides (A–D) and the genomic DNA of *S. aureus* (E, F). (A) Fluorescence recovery percentage (%) of TALE tetM_1298 (*p < 0.0001*). (B) ADNA-dose-dependent plot of TALE tetM_1298. (C) Fluorescence recovery percentage (%) of TALE tetM_611 (*p < 0.0001*). (D) A DNA-dose-dependent plot of TALE tetM_611. Limit of detection of (E) TALE tetM_1298 and (F) TALE tetM_611 (*p < 0.05*).

Fluorescence-based detection methods have a wide range of applications in many studies of biochemistry and biomedicine.^19,32^ In our previous study, we developed a detection method using DNA-binding zinc finger proteins (ZFPs) with a size of 65 kDa, smaller than 120 kDa TALEs that provided a limit of detection of 1 nM.^20^ Considering that the height of QD-labeled TALEs was approximately 20 nm (Figure 3C), traveling even a short distance from the GO surface would easily extend the distance between TALEs and GO to be greater than 30 nm, resulting in more restoration of the signal and increased sensitivity. Thus, we were able to develop an improved detection method by utilizing novel TALEs. Our detection system with TALEs was able to recognize its target DNA sequence at much lower concentrations such as 1 pM or 1 fM, as shown in Figure 5. The percent recovery of the fluorescence signal was calculated using eq 2 where F_0_ and F_1_ are the fluorescence intensity at the maxima in the absence and presence of GO and F_i_ is the fluorescence intensity at the maxima in the presence of target DNA.
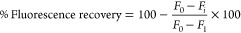
2

The TALE tetM_1298 and tetM_611 were designed to recognize 14 and 16 base pairs, respectively, to compare the detection efficiency of the two TALEs recognizing the different lengths of the target DNA. As shown in Figure 5A–D, the signal was restored quantitatively as DNA concentrations increased from 1 pM to 100 nM. The limit of detection of TALE tetM_1298 and tetM_611 was determined to be 10 pM (*p < 0.0001*) and 1 pM (*p < 0.0001*), respectively, in the presence of target DNA oligonucleotides based on the statistical analysis of the sensitivity data with ANOVA (one-way analysis of variance), as shown in Figure 5A, C. As TALE tetM_611 binds to 16 bp DNA having two more repeats compared to TALE tetM_1298, the increased number of TALE repeats could contribute to the stability of the TALE and DNA bound complex,^33^ resulting in higher sensitivity. This result can indicate that an increase in the number of repeats in TALEs could contribute to improved sensitivity.

We also investigated if our sensing system is able to recognize the target sites of 14 and 16 bp in the presence of the complex genomic DNA. The genomic DNA of *Staphylococcus aureus* containing the *tetM* gene was used instead of DNA oligonucleotides. As shown in Figure 5F, the limit of detection of TALE tetM_611 was determined to be 1 fM (*p < 0.05*). The average recovery percentage of TALE tetM_611 was 4 and 10% at 1 and 10 fM, respectively. However, the average recovery percentage of TALE tetM_1298 was increased by only 1% from 1 to 10 fM (Figure 5E), which was not significantly different. Thus, the limit of detection for TALE tetM_1298 was determined to be 10 fM. This result of the genomic DNA is also in good agreement with that of DNA oligonucleotides, demonstrating that TALE tetM_611 showed higher sensitivity than TALE tetM_1298. Our TALE-GO-FRET-based sensing system was able to detect their cognate DNA sites in the presence of the complex genomic DNA which can act as millions of nonspecific DNAs. In addition, the limit of detection with genomic DNA was lower than that with DNA oligonucleotides. As the surface of GO contains carboxyl groups that are deprotonated at a neutral pH and DNA is a polyanion, we expect that some of the DNA molecules might be repelled by the negatively charged GO due to electrostatic repulsion between them.^34^ Length-dependent interaction of DNA with GO was previously investigated and the study demonstrated that shorter DNA was more effectively loaded on GO surface.^34^ In other words, the larger amount of polyanions present in longer DNA might contribute to increased repulsion between DNA and GO, resulting in a higher restoration signal and a lower limit of detection. This might explain the prompt dissociation from GO when TALEs are bound to genomic DNA.^35−37^ Based on the target DNA sequence comparison, the DNA sequence of TALE tetM_611 has one less guanine base than that of TALE tetM_1298, which might potentially influence the desorption of DNA from the GO surface as the guanine base is reported to bind to graphene more strongly than adenine, thymine, and cytosine.^38^ This might slightly affect more dissociation from GO when TALE tetM_611 was bound to the target DNA. Taken together, our TALE-GO-FRET-based sensing system was able to detect their cognate DNA sites at 1 fM of genomic DNA concentration. Therefore, we expect that our system has the potential for application with real-world biological samples such as cell lysate.

Some proteins, salt ions, and organic compounds that may be present in the real-world biological sample might be potential competitors for the adsorption of TALEs on the GO. However, adsorption could be a complex process where factors such as pH, concentration, and pI of proteins could affect the adsorption efficiency.^39^ Thus, it is unknown that these potential competitors could indeed efficiently adsorb to the GO under our assay conditions. Also, TALEs (120 kDa) are much bigger than some of the proteins, ions, and organic compounds in the biological sample, resulting in larger surface areas available to interact with GO. Thus, TALEs could interact more strongly with GO via multiple noncovalent interactions such as electrostatic interactions, π – π stacking, and hydrogen bonding, compared to the potential competitors described earlier. Additionally, these relatively small molecules of potential competitors could be removed quickly and simply using a filtration unit before applying the sample to the sensing system.

Loop-mediated isothermal amplification (LAMP) has its merits such as high specificity and simpler hardware requirements as compared to PCR. When it was integrated with microfluidics, an LOD of 1 copy/μL of λDNA was achieved.^40,41^ Currently, our detection method is not as sensitive as this method as our system is not integrated with a microfluidic and our approach is different from nucleic acid amplification methods such as PCR and LAMP. However, it should be noted that our GO-based method runs at room temperature without requiring careful control of temperature whereas LAMP-based methods still need an elevated temperature of ∼65 °C. Moreover, our GO-based assay is a one-pot system by simply incubating GO, TALEs, and dsDNA, which does not need the expensive reagents required for PCR. Integration of our system into a microfluidic module could be investigated in future studies to further improve the sensitivity.

### Specificity

**Figure 6:**
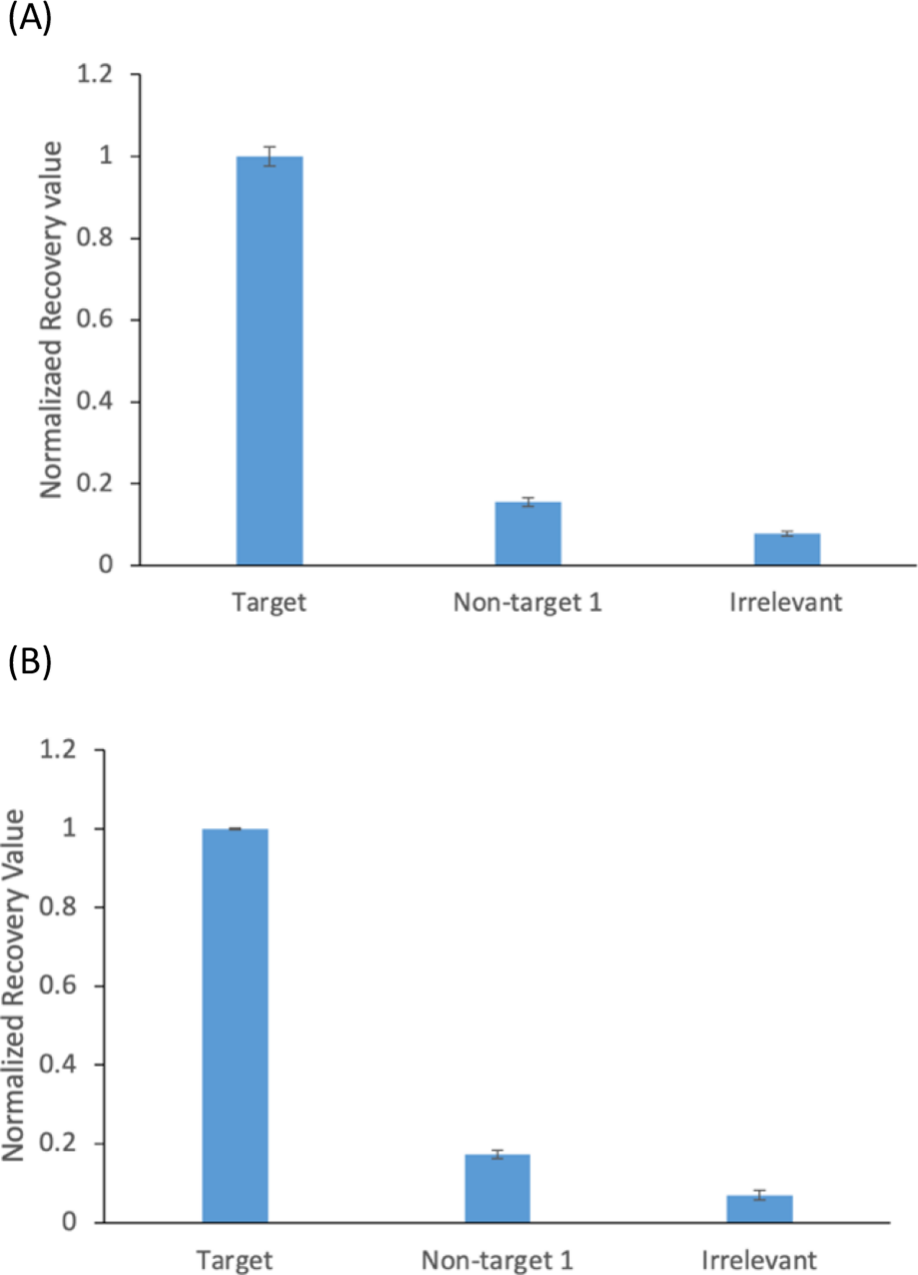
Specificity of TALE (A) tetM_1298 and (B) tetM_611. TALEs are incubated with their own target DNA as well as nontarget and irrelevant DNA.

We demonstrated that our detection method can distinguish between target and nontarget sequences by using different DNA oligonucleotides such as the target, nontarget, and irrelevant DNAs. Their sequences are provided in Supporting Information (Table S2). The target and nontarget sequences are located in two different regions in the *tetM* gene (Figure S2). The irrelevant sequence is not present in the genome of *E. coil* nor in that of *S. aureus*. The nontarget sequences of oligonucleotides are matched with their target sequences at 21 and 18% for tetM_1298 and tetM_611, respectively (Table S2). As shown in Figure 6, both TALE tetM_1298 and tetM_611 were still able to recognize their cognate DNA with a significantly higher signal as compared to nontarget and irrelevant DNAs. Thus, our engineered TALEs were able to distinguish their own target DNA from nontarget and irrelevant DNA showing high specificity.

## Conclusions

In this study, we developed a rapid and sensitive detection system for antibiotic-resistant dsDNA by integrating novel DNA-binding domain TALEs and 2D nanosheet GO. Our detection method is expected to produce a signal in the presence of the target DNA via FRET. Thus, the adsorption of QD-labeled TALEs on the GO surface is vital to quench the signal by GO. To the best of our knowledge, we demonstrated for the first time that TALEs could immobilize on the surface of GO. Upon target DNA binding, engineered TALEs would dissociate from the GO surface, resulting in a high restoration signal and improved sensitivity as low as 1 fM compared to ZFPs. TALEs can directly bind the dsDNA and rapidly search for the target site along the dsDNA after a short incubation time of 10 min, allowing us to avoid the laborious steps of DNA denaturation and subsequent hybridization involved in PCR.^8^ In addition, our approach is able to detect multiple dsDNA targets via labeling different colored QDs onto TALEs engineered to recognize multiple different antibiotic resistance genes. In future studies, we will focus on developing the application of real-world biological samples. We envision that our detection method has great potential for a rapid and sensitive point-of-care application.

## Supporting Information Available

SDS-PAGE gel of purified TALEs; the location of the target region in the *tetM* gene; peptide sequences in TALEs; sequences of the target, nontarget, and irrelevant DNA; sequences of EMSA oligonucleotides; and illustration of EMSA (PDF)

## References

[ref1] Miranda C. D.; Kehrenberg C.; Ulep C.; Schwarz S.; Roberts M. C. Diversity of tetracycline resistance genes in bacteria from Chilean salmon farms. Antimicrob. Agents Chemother. 2003, 47, 883–888. 10.1128/AAC.47.3.883-888.2003. 12604516 PMC149303

[ref2] Roberts M. C. Update on acquired tetracycline resistance genes. FEMS Microbiol. Lett. 2005, 245, 195–203. 10.1016/j.femsle.2005.02.034. 15837373

[ref3] McKinney C. W.; Dungan R. S.; Moore A.; Leytem A. B. Occurrence and abundance of antibiotic resistance genes in agricultural soil receiving dairy manure. FEMS Microbiol. Ecol. 2018, 94 ( (3), ), 10.1093/femsec/fiy010. 29360961

[ref4] Tzimoula K.; Maria E.; Eudoxia D.; Georgia G.; Angeliki M.; Nikolaos M. Detection of the tetM resistance determinant among phenotypically sensitive Ureaplasma species by a novel real-time PCR method. Diagn. Microbiol. Infect. Dis. 2015, 81, 85–88. 10.1016/j.diagmicrobio.2014.10.013. 25467173

[ref5] Zhu D. K.; Luo H. Y.; Liu M. F.; Zhao X. X.; Jia R. Y.; Chen S.; Sun K. F.; Yang Q.; Wu Y.; Chen X. Y.; Cheng A. C.; Wang M. S. Various Profiles of tet Genes Addition to tet(X) in Riemerella anatipestifer Isolates From Ducks in China. Front. Microbiol. 2018, 9, 585 10.3389/fmicb.2018.00585. 29636748 PMC5880999

[ref6] Luby E.; Ibekwe A. M.; Zilles J.; Pruden A. Molecular Methods for Assessment of Antibiotic Resistance in Agricultural Ecosystems: Prospects and Challenges. J. Environ. Qual. 2016, 45, 441–453. 10.2134/jeq2015.07.0367. 27065390

[ref7] Boch J.; Bonas U. Xanthomonas AvrBs3 family-type III effectors: discovery and function. Annu. Rev. Phytopathol. 2010, 48, 419–436. 10.1146/annurev-phyto-080508-081936. 19400638

[ref8] Cuculis L.; Abil Z.; Zhao H.; Schroeder C. M. TALE proteins search DNA using a rotationally decoupled mechanism. Nat. Chem. Biol. 2016, 12, 831–837. 10.1038/nchembio.2152. 27526029

[ref9] Deng D.; Yan C.; Pan X.; Mahfouz M.; Wang J.; Zhu J. K.; Shi Y.; Yan N. Structural basis for sequence-specific recognition of DNA by TAL effectors. Science 2012, 335, 720–723. 10.1126/science.1215670. 22223738 PMC3586824

[ref10] Boch J.; Scholze H.; Schornack S.; Landgraf A.; Hahn S.; Kay S.; Lahaye T.; Nickstadt A.; Bonas U. Breaking the code of DNA binding specificity of TAL-type III effectors. Science 2009, 326, 1509–1512. 10.1126/science.1178811. 19933107

[ref11] Mak A. N.; Bradley P.; Cernadas R. A.; Bogdanove A. J.; Stoddard B. L. The crystal structure of TAL effector PthXo1 bound to its DNA target. Science 2012, 335, 716–719. 10.1126/science.1216211. 22223736 PMC3427646

[ref12] Mak A. N.; Bradley P.; Bogdanove A. J.; Stoddard B. L. TAL effectors: function, structure, engineering and applications. Curr. Opin. Struct. Biol. 2013, 23, 93–99. 10.1016/j.sbi.2012.11.001. 23265998 PMC3572262

[ref13] Chaudhary K.; Kumar K.; Venkatesu P.; Masram D. T. Protein immobilization on graphene oxide or reduced graphene oxide surface and their applications: Influence over activity, structural and thermal stability of protein. Adv. Colloid Interface Sci. 2021, 289, 102367 10.1016/j.cis.2021.102367. 33545443

[ref14] Pena-Bahamonde J.; Nguyen H. N.; Fanourakis S. K.; Rodrigues D. F. Recent advances in graphene-based biosensor technology with applications in life sciences. J. Nanobiotechnol. 2018, 16, 75 10.1186/s12951-018-0400-z. PMC6150956 30243292

[ref15] Bei H. P.; Yang Y.; Zhang Q.; Tian Y.; Luo X.; Yang M.; Zhao X. Graphene-Based Nanocomposites for Neural Tissue Engineering. Molecules 2019, 24, 658 10.3390/molecules24040658. 30781759 PMC6413135

[ref16] Zhang J. L.; Zhang F.; Yang H. J.; Huang X. L.; Liu H.; Zhang J. Y.; Guo S. W. Graphene Oxide as a Matrix for Enzyme Immobilization. Langmuir 2010, 26, 6083–6085. 10.1021/la904014z. 20297789

[ref17] Zhou L.; Jiang Y.; Gao J.; Zhao X.; Ma L. Graphene oxide as a matrix for the immobilization of glucose oxidase. Appl. Biochem. Biotechnol. 2012, 168, 1635–1642. 10.1007/s12010-012-9884-4. 22965306

[ref18] Zou X. Q.; Wei S.; Jasensky J.; Xiao M. Y.; Wang Q. M.; Brooks C. L.; Chen Z. Molecular Interactions between Graphene and Biological Molecules. J. Am. Chem. Soc. 2017, 139, 1928–1936. 10.1021/jacs.6b11226. 28092440

[ref19] Zheng P.; Wu N. Fluorescence and Sensing Applications of Graphene Oxide and Graphene Quantum Dots: A Review. Chem. – Asian J. 2017, 12, 2343–2353. 10.1002/asia.201700814. 28742956 PMC5915373

[ref20] Ha D. T.; Nguyen V. T.; Kim M. S. Graphene Oxide-Based Simple and Rapid Detection of Antibiotic Resistance Gene via Quantum Dot-Labeled Zinc Finger Proteins. Anal. Chem. 2021, 93, 8459–8466. 10.1021/acs.analchem.1c00560. 34097379

[ref21] Nguyen T. V. T.; Le B. H.; Seo Y. J. Highly fluorescence quenching graphene oxide-based oligodeoxynucleotide hairpin systems for probing CNG DNA repeat sequences. Tetrahedron Lett. 2017, 58, 3301–3305. 10.1016/j.tetlet.2017.07.035.

[ref22] Kasry A.; Ardakani A. A.; Tulevski G. S.; Menges B.; Copel M.; Vyklicky L. Highly Efficient Fluorescence Quenching with Graphene. J. Phys. Chem. C 2012, 116, 2858–2862. 10.1021/jp207972f.

[ref23] Qaddare S. H.; Salimi A. Amplified fluorescent sensing of DNA using luminescent carbon dots and AuNPs/GO as a sensing platform: A novel coupling of FRET and DNA hybridization for homogeneous HIV-1 gene detection at femtomolar level. Biosens. Bioelectron. 2017, 89, 773–780. 10.1016/j.bios.2016.10.033. 27816581

[ref24] Zhang Z.; Liu Y.; Ji X.; Xiang X.; He Z. A graphene oxide-based enzyme-free signal amplification platform for homogeneous DNA detection. Analyst 2014, 139, 4806–4809. 10.1039/C4AN00933A. 25058563

[ref25] Shi J.; Guo J.; Bai G.; Chan C.; Liu X.; Ye W.; Hao J.; Chen S.; Yang M. A graphene oxide based fluorescence resonance energy transfer (FRET) biosensor for ultrasensitive detection of botulinum neurotoxin A (BoNT/A) enzymatic activity. Biosens. Bioelectron. 2015, 65, 238–244. 10.1016/j.bios.2014.10.050. 25461164

[ref26] Dong H.; Gao W.; Yan F.; Ji H.; Ju H. Fluorescence resonance energy transfer between quantum dots and graphene oxide for sensing biomolecules. Anal. Chem. 2010, 82, 5511–5517. 10.1021/ac100852z. 20524633

[ref27] Kim M. S.; Kini A. G. Engineering and Application of Zinc Finger Proteins and TALEs for Biomedical Research. Mol. Cells 2017, 40, 533–541. 10.14348/molcells.2017.0139. 28835021 PMC5582299

[ref28] He X.; Ma N. A general strategy for label-free sensitive DNA detection based on quantum dot doping. Anal. Chem. 2014, 86, 3676–3681. 10.1021/ac500590d. 24627946

[ref29] Bartczak D.; Kanaras A. G. Preparation of peptide-functionalized gold nanoparticles using one pot EDC/sulfo-NHS coupling. Langmuir 2011, 27, 10119–10123. 10.1021/la2022177. 21728291

[ref30] Huang A.; Zhang L.; Li W.; Ma Z.; Shuo S.; Yao T. Controlled fluorescence quenching by antibody-conjugated graphene oxide to measure tau protein. R. Soc. Open Sci. 2018, 5, 171808 10.1098/rsos.171808. 29765647 PMC5936912

[ref31] von Hippel P. H. From “simple” DNA-protein interactions to the macromolecular machines of gene expression. Annu. Rev. Biophys. Biomol. Struct. 2007, 36, 79–105. 10.1146/annurev.biophys.34.040204.144521. 17477836 PMC2660389

[ref32] Liu F.; Yang Y.; Wan X.; Gao H.; Wang Y.; Lu J.; Xu L. P.; Wang S. Space-Confinment-Enhanced Fluorescence Detection of DNA on Hydrogel Particles Array. ACS Nano 2022, 16, 6266–6273. 10.1021/acsnano.2c00157. 35385247

[ref33] Geiger-Schuller K.; Mitra J.; Ha T.; Barrick D. Functional instability allows access to DNA in longer transcription Activator-Like effector (TALE) arrays. Elife 2019, 8, e38298 10.7554/eLife.38298. 30810525 PMC6461438

[ref34] Wu M.; Kempaiah R.; Huang P. J.; Maheshwari V.; Liu J. Adsorption and desorption of DNA on graphene oxide studied by fluorescently labeled oligonucleotides. Langmuir 2011, 27, 2731–2738. 10.1021/la1037926. 21302946

[ref35] Zeng S. W.; Chen L.; Wang Y.; Chen J. L. Exploration on the mechanism of DNA adsorption on graphene and graphene oxide via molecular simulations. J. Phys. D: Appl. Phys. 2015, 48, 275402 10.1088/0022-3727/48/27/275402.

[ref36] Bi S.; Zhao T.; Luo B. A graphene oxide platform for the assay of biomolecules based on chemiluminescence resonance energy transfer. Chem. Commun. 2012, 48, 106–108. 10.1039/C1CC15443E. 22037540

[ref37] Huang P. J.; Liu J. Separation of Short Single- and Double-Stranded DNA Based on Their Adsorption Kinetics Difference on Graphene Oxide. Nanomaterials 2013, 3, 221–228. 10.3390/nano3020221. 28348332 PMC5327888

[ref38] Varghese N.; Mogera U.; Govindaraj A.; Das A.; Maiti P. K.; Sood A. K.; Rao C. N. Binding of DNA nucleobases and nucleosides with graphene. ChemPhysChem 2009, 10, 206–210. 10.1002/cphc.200800459. 18814150

[ref39] Gao Y.; Li Y.; Zhang L.; Huang H.; Hu J. J.; Shah S. M.; Su X. G. Adsorption and removal of tetracycline antibiotics from aqueous solution by graphene oxide. J. Colloid Interface Sci. 2012, 368, 540–546. 10.1016/j.jcis.2011.11.015. 22138269

[ref40] Mao P.; Cao L.; Li Z.; You M.; Gao B.; Xie X.; Xue Z.; Peng P.; Yao C.; Xu F. A digitalized isothermal nucleic acid testing platform based on a pump-free open droplet array microfluidic chip. Analyst 2021, 146, 6960–6969. 10.1039/D1AN01373D. 34657942

[ref41] Cao L.; Guo X.; Mao P.; Ren Y.; Li Z.; You M.; Hu J.; Tian M.; Yao C.; Li F.; Xu F. A Portable Digital Loop-Mediated Isothermal Amplification Platform Based on Microgel Array and Hand-Held Reader. ACS Sens. 2021, 6, 3564–3574. 10.1021/acssensors.1c00603. 34606243

