# Supplementary material for: Rapid and Sensitive Detection of Antibiotic Resistance Genes by Utilizing TALEs as a Diagnostic Probe with 2D-Nanosheet Graphene Oxide
Source: Anal Chem. 2023 Jun 13;95(25):9505–12. doi: 10.1021/acs.analchem.3c00647 (PMC10308326; doi:10.1021/acs.analchem.3c00647)
Supplement: Supplementary file 1 — ac3c00647_si_001.pdf [file ac3c00647_si_001.pdf]

Supplementary

Rapid and sensitive detection of antibiotic resistance genes by utilizing TALEs a diagnostic probe with 2D-nanosheet graphene oxide

Jihye Kang, Van-Thuan Nguyen, and Moon-Soo Kim*

Department of Chemistry, Western Kentucky University, Bowling Green, Kentucky 42101, USA

Table of content

Figure S1. The SDS PAGE gels show the purity and the expected molecular weight of TALEs. (A) TALE_tetM611 and (B) TALE_tetM1298.

Figure S2. Location of the target regions of TALE tetM_1298 and tetM_611 in the *tetM* gene and selected non-target sequences of tetM_1298 and tetM_611.

Table S1. The composition of amino acid sequences in the TALEs.

Table S2. Sequences of the target, non-target, and irrelevant oligonucleotides for TALE tetM_1298 and tetM_611.

Table S3. The sequence of EMSA oligonucleotides for TALE tetM_1298 and tetM_611.

Figure S3. Illustration of EMSA (electromobility shift assay) of (A) TALE tetM_1298 and (B) TALE tetM_611. The TALE concentrations (nM) are given above the gel image. The top bands indicate a bound complex of TALEs and DNA and the bottom bands show free DNA.

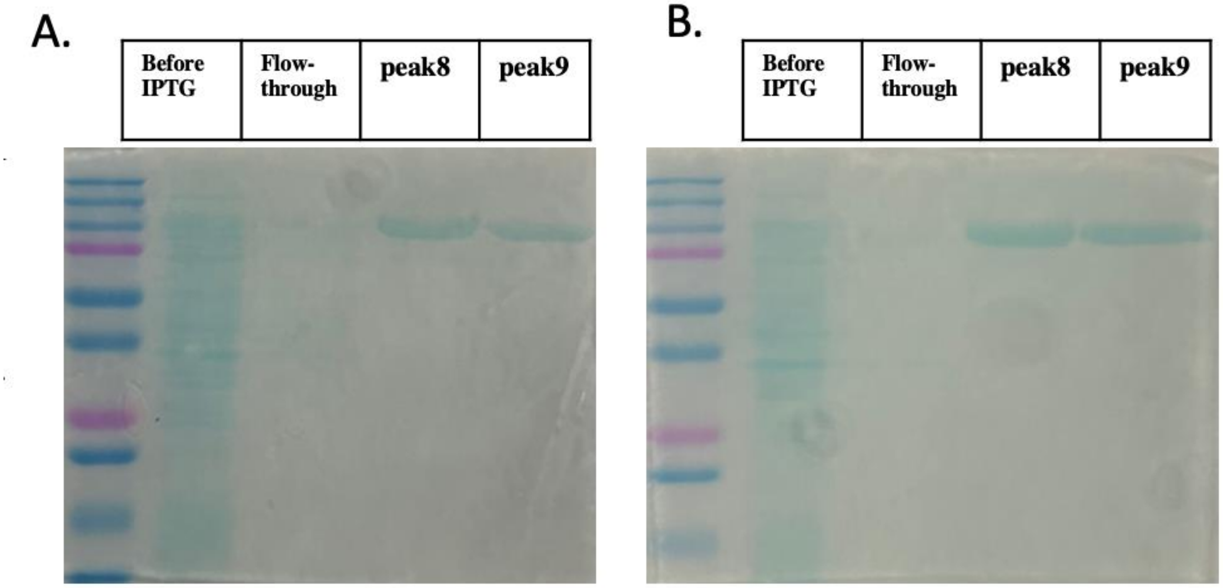

Figure S1. The SDS-PAGE gels show the purity and the expected molecular weight of (A) TALE tetM_611 and (B) TALE tetM_1298.

Staphylococcus aureus strain 2952 tetracycline resistance protein TetM (tetM) gene, partial cds

GenBank: AY057894.1

AGTTTTAGCTCATGTTGATGCGGGAAAAACTACCTTAACAGAAAGCTTATTATATAACAGTGGAG
CGATTACAGAATTAGGAAGCGTGGACAAAGGTACAACGAGGACGGATAATACGCTTTTAGAACG
TCAGAGAGGAATTACAATTCAGACAGGAATAACCTCTTTTCAGTGGGAAAATACGAAGGTGAAC
ATCATAGACACGCCAGGACATATGGATTTCTTAGCAGAAGTATATCGTTCATTATCAGTTTTAGA
TGGGGCAATTCTACTGATTTCTGCAAAAGATGGCGTACAAGCACAAACTCGTATATTATTTCATG
CACTTAGGAAAATGGGGATTCCCACAATCTTTTTTATCAATAAGATTGACCAAAATGGAATTGAT
TTATCAACGGTTTATCAGGATATTAAAGAGAAACTTTCTGCCGAAATTGTAATCAAACAGAAGGT
AGAACTGTATCCTAATGTGTGTGTGACGAACTTTACCGAATCTGAACAATGGGATACGGTAATAG
AGGGAAACGATGACCTTTTAGAGAAATATATGTCCGGTAAATCATTAGAAGCATTGGAACTCGA
ACAAGAGGAAAGCATAAGATTTCAGAATTGTTCTCTGTTCCCTCTTTATCATGGAAGTGCAAAAA
GTAATATAGGGATTGATAACCTTATAGAAGTGATTACGAATAAATTTTATTCATCAACACATCGA
GGTCAGTCTGAACTTTGCGGAAATGTTTTCAAAATTGAATATACAAAAAAAAGACAACGTCTTGC
ATATATACGTCTTTATAGTGGCGTACTGCATTTGCGAGATTCGGTTAGAATATCGGAAAAGGAAA
AAATAAAAATTACAGAAATGTATACTTCAATAAATGGTGAATTATGTAAAATCGATAAGGCTTAT
TCCGGGGAAATTGTTATTTTGCAAAATGAGTTTTTGAAGTTAAATAGTGTTCTTGGAGATACAAA
ACTATTGCCACAGAGAAAAAAGATTGAAAATCCGCACCCTCTACTACAAACAACTGTTGAACCG
AGTAAACCTGAACAGAGAGAAATGTTGCTTGATGCCCTTTTGGAAATCTCAGATAGTGATCCGCT
TCTACGATATTACGTGGATTCTACGACACATGAAATTATACTTTCTTTCTTAGGGAAAGTACAAAT
GGAAGTGATTAGTGCACTGTTGCAAGAAAAGTATCATGTGGAGATAGAACTAAAAGAGCCTACA
GTCATTTATATGGAGAGACCGTTAAAAAATGCAGAATATACCATTCACATCGAAGTGCCGCCAAA
TCCTTTCTGGGCTTCCATTGGTTTATCTGTATCACCGCTTCCGTTGGGAAGTGGAATGCAGTATGA
GAGCTCGGTTTCTCTTGGATACTTAAATCAATCATTTCAAAATGCAGTTATGGAAGGGATACGCT
ATGGCTGTGAACAAGGATTGTATGGTTGGAATGTGACGGACTGTAAAATCTGTTTTAAGTATGGC
TTATACTATAGCCCTGTTAGTACCCCAGCAGATTTTCGGATGCTTGCTCCTATTGTATTGGAACAA
GTCTTAAAAAAAGCTGGAACAGAATTGTTAGAGCCATATCTTAGTTTTAAAATTTATGCGCCACA
GGAATATCTTTCACGAGCATACAACGATGCTCCTAAATATTGTGCGAACATCGTAGACACTCAAT
TGAAAAATAATGAGGTCATTCTTAGTGGAGAAATCCCTGCTCGGTGTATTCAAGAATATCGTAGT
GATTTAACTTTCTTTACAAATGGACGTAGTGTTTGTTTAACAGAGTTAAAAGGGTACCATGTTACT
ACCGGTGAACCTGTTTGCCAGCCCCGTCGTCTAAATAGTCGGA

Blue color: TALE tetM_1298 target sequence
Yellow color: TALE tetM_1298 non-target sequence

Violet color: TALE tetM_611 target sequence
Green color: TALE tetM_611 non-target sequence

Figure S2. Location of the target regions of tetM_1298 and tetM_611 in the *tetM* gene and selected non-target sequences of tetM_1298 and tetM_611.

Table S1. The composition of amino acid sequences in TALEs.

**TALE tetM_1298**		Percentage	Number
Aromatic ring	Phe	1.6%	17
	Trp	0.9%	10
	Tyr	1.7%	18
	His	3.6%	38
Amide side chain	Asn	4.6%	49
	Gln	7%	75
	Arg	3%	32
Hydrophilic side chain	Lys	5.3%	57

**TALE tetM_611**		Percentage	Number
Aromatic ring	Phe	1.5%	17
	Trp	0.9%	10
	Tyr	1.6%	18
	His	3.6%	41
Amide side chain	Asn	4.3%	49
	Gln	7.3%	83
	Arg	3%	34
Hydrophilic side chain	Lys	5.2%	59

Table S2. Sequences of the target, non-target, and irrelevant oligonucleotides for TALE tetM_1298 and tetM_611.

5'-G-target sequence-G-3'
3'-C-complementary of target sequence-C-5'

The nucleotide sequences that match the target sequence are highlighted in red.

A) tetM_1298 target

	Sequence	Length (bp)	GC%	Percentage of sequence similarity with the target sequence (red color)
Forward	5'-GGCTCCTTTCTGGGCTTC GGG-3'	21	66.7	-
Reverse	5'-CCCGAAGCCCAGAAAGGAGCC-3'	21	66.7	-

B) tetM_1298 non-target

	Sequence	Length (bp)	GC%	Percentage of sequence similarity with the target sequence (red color)
Forward	5'-GTTAACAGAAAGCTTG-3'	16	37.5	21%
Reverse	5'-CAAG CTT TCT GTTAAC-3'	16	37.5	0%

C) tetM_1298 irrelevant

	Sequence	Length (bp)	GC%	Percentage of sequence similarity with the target sequence (red color)
Forward	5'-GTGTC AGG GCC CTC GATG-3'	18	66.7	0%
Reverse	5'-ACTCG AGG GCC CTG ACAC-3'	18	66.7	0%

D) tetM_611 target

	Sequence	Length (bp)	GC%	Percentage of sequence similarity with the target sequence (red color)
Forward	5'-GGCTTGTTCTCTGTTCCCTCGGG-3'	23	60.9	-
Reverse	5'-CCCGAGGGAACAGAGAACAAGC C-3'	23	60.9	-

E) tetM_611 non-target

	Sequence	Length (bp)	GC%	Percentage of sequence similarity with the target sequence (red color)
Forward	5’-GGCGATGCGGGAAAA ACTACGGG-3’	23	60.9	18%
Reverse	5’-CCCGTAGTTTTT CCCGCATCGCC-3’	23	60.9	0%

F) tetM_611 irrelevant

	Sequence	Length (bp)	GC%	Percentage of sequence similarity with the target sequence (red color)
Forward	5’-GCGCACTGTATACTTGAGCG -3’	20	55	0%
Reverse	5’-CGCTCAAGTATACAGTG CGC-3’	20	55	0%

Table S3. Sequences of EMSA oligonucleotides for TALE tetM_1298 and tetM_611.

A) tetM_1298

	Sequence	Length (bp)	GC%
Forward	5'-biotin/GCGCCTTTCTGGGCTTCGCG-3'	20	70%
Reverse	5'-TTTTTTTTTTTTTTTCGCGAAGCCCAG AAAGGCGC-3'	35	40%

B) tetM_611

	Sequence	Length (bp)	GC%
Forward	5'-biotin/GCGTGTTCTCTGTTCCCTCGCG-3'	22	63.6%
Reverse	5'-TTTTTTTTTTTTTTTCGCGAGGGAACA GAGAACACGC-3'	37	37.8%

A) tetM_1298

200	85.6	36.63	15.5	6.71	2.87	1.22	0.52	0.23	0	nM

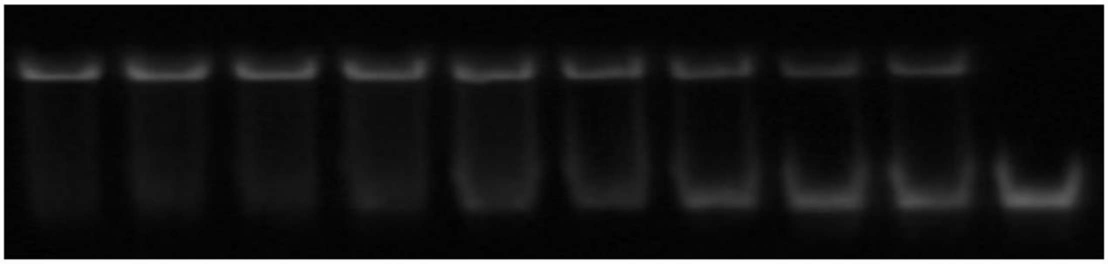

B) tetM_611

25	10.7	4.6	1.96	0.83	0.36	0.15	0.07	0.03	0	nM

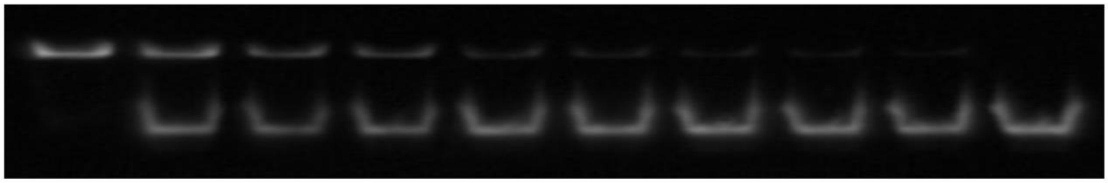

Figure S3. Illustration of EMSA (electromobility shift assay) of (A) TALE tetM_1298 and (B) TALE tetM_611. The TALE concentrations (nM) are given above the gel image. The top bands indicate a bound complex of TALEs and DNA and the bottom bands show free DNA.
